# An interactive videogame designed to improve respiratory navigator efficiency in children undergoing cardiovascular magnetic resonance

**DOI:** 10.1186/s12968-016-0272-z

**Published:** 2016-09-06

**Authors:** Sean M. Hamlet, Christopher M. Haggerty, Jonathan D. Suever, Gregory J. Wehner, Jonathan D. Grabau, Kristin N. Andres, Moriel H. Vandsburger, David K. Powell, Vincent L. Sorrell, Brandon K. Fornwalt

**Affiliations:** 1 Department of Electrical Engineering, University of Kentucky, Lexington, KY USA; 2 Department of Pediatrics, University of Kentucky, Lexington, KY USA; 3 Institute for Advanced Application, Geisinger Health System, Danville, PA USA; 4 Department of Biomedical Engineering, University of Kentucky, Lexington, KY USA; 5 Department of Physiology, University of Kentucky, Lexington, KY USA; 6 Department of Medicine, University of Kentucky, Lexington, KY USA

**Keywords:** Pediatrics, Respiratory navigator, Navigator efficiency, Image quality, Cardiovascular magnetic resonance

## Abstract

**Background:**

Advanced cardiovascular magnetic resonance (CMR) acquisitions often require long scan durations that necessitate respiratory navigator gating. The tradeoff of navigator gating is reduced scan efficiency, particularly when the patient’s breathing patterns are inconsistent, as is commonly seen in children. We hypothesized that engaging pediatric participants with a navigator-controlled videogame to help control breathing patterns would improve navigator efficiency and maintain image quality.

**Methods:**

We developed custom software that processed the Siemens respiratory navigator image in real-time during CMR and represented diaphragm position using a cartoon avatar, which was projected to the participant in the scanner as visual feedback. The game incentivized children to breathe such that the avatar was positioned within the navigator acceptance window (±3 mm) throughout image acquisition.

Using a 3T Siemens Tim Trio, 50 children (Age: 14 ± 3 years, 48 % female) with no significant past medical history underwent a respiratory navigator-gated 2D spiral cine displacement encoding with stimulated echoes (DENSE) CMR acquisition first with no feedback (NF) and then with the feedback game (FG). Thirty of the 50 children were randomized to undergo extensive off-scanner training with the FG using a MRI simulator, or no off-scanner training. Navigator efficiency, signal-to-noise ratio (SNR), and global left-ventricular strains were determined for each participant and compared.

**Results:**

Using the FG improved average navigator efficiency from 33 ± 15 to 58 ± 13 % (*p* < 0.001) and improved SNR by 5 % (*p* = 0.01) compared to acquisitions with NF. There was no difference in navigator efficiency (*p* = 0.90) or SNR (*p* = 0.77) between untrained and trained participants for FG acquisitions. Circumferential and radial strains derived from FG acquisitions were slightly reduced compared to NF acquisitions (−16 ± 2 % vs −17 ± 2 %, *p* < 0.001; 40 ± 10 % vs 44 ± 11 %, *p* = 0.005, respectively). There were no differences in longitudinal strain (*p* = 0.38).

**Conclusions:**

Use of a respiratory navigator feedback game during navigator-gated CMR improved navigator efficiency in children from 33 to 58 %. This improved efficiency was associated with a 5 % increase in SNR for spiral cine DENSE. Extensive off-scanner training was not required to achieve the improvement in navigator efficiency.

**Electronic supplementary material:**

The online version of this article (doi:10.1186/s12968-016-0272-z) contains supplementary material, which is available to authorized users.

## Background

Cardiovascular magnetic resonance (CMR) can be used to non-invasively assess heart function. In the clinical setting, CMR techniques play an important role in the diagnosis and monitoring of the complex anatomy and physiology of congenital and acquired heart diseases. Moreover, there is a considerable body of pre-clinical research devoted to the development and evaluation of new, advanced imaging techniques, such as 3D displacement encoding with stimulated echoes (DENSE) [1], 3D steady state free precession [2], and 4D flow imaging [3]. These new techniques have demonstrated ability in distinguishing normal and pathological tissue deformation and blood flow and may become beneficial tools in the diagnosis and management of heart disease. Many of these clinical and pre-clinical techniques require scan durations that exceed patients’ ability to hold their breath.

**Fig. 1:**
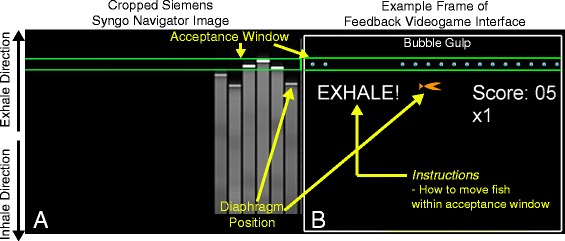
Feedback videogame. **a** Cropped version of the Siemens Syngo navigator image that was processed in real-time during CMR acquisition to yield the feedback videogame. **b** Example frame of the navigator feedback videogame interface, which was shown to the child during CMR (yellow overlay text was not shown to the child)

End-expiratory breath-holds are used by many CMR sequences in order to minimize respiratory-motion artifacts. However, requiring subjects to hold their breath introduces significant limitations on the duration of data acquisition or the quality of the acquired images, particularly for young children or patients with advanced disease. A common alternative is respiratory navigator gating, which works by measuring the diaphragm position during normal breathing and only acquiring data when the diaphragm is within a pre-defined acceptance window (Fig. 1a). The trade-off of navigator gating is significantly increased scan duration because of poor navigator efficiency. For example, previous CMR studies have reported respiratory navigator efficiencies of 20 to 45 % in adults [4–7]. This poor navigator efficiency lengthens the duration of currently used clinical imaging and limits clinical feasibility of emerging advanced imaging techniques.

Navigator efficiency is typically poor because breathing patterns can be erratic [8–10] and the patient is generally unaware of the desired acceptance window location. Providing the patient with visual feedback of the diaphragm position during CMR (“navigator feedback”) has been shown to improve breathing consistency and scan efficiency in adults [5, 8]. For example, studies have shown efficiency improvements up to 29 % (absolute) compared to traditional acquisitions without feedback [5, 6]. Importantly, these previous studies have demonstrated that image quality from navigator feedback acquisitions is similar to acquisitions without feedback [5, 6]. The potential to achieve similar benefits using navigator feedback with pediatric participants has not been explored. Given the challenge of keeping these participants still and motionless for long periods of time, this improved efficiency could have substantial clinical benefit.

Most previous studies involving navigator feedback simply utilized the built-in navigator display. One previous study evaluated a custom videogame interface in a study of adults for increasing navigator efficiency [6]. Such an interface theoretically combines the benefits of visual feedback with an intuitive and engaging design for the user—attributes that are highly desirable for pediatric scanning. Thus, the present study sought to extend and tailor this paradigm specifically for children by providing navigator feedback in the form of an interactive, kid-friendly videogame. Moreover, this study sought to test this design using DENSE, an imaging technique that can be used to quantify advanced measures of function such as cardiac strains. We hypothesized that navigator feedback using an interactive videogame during CMR would improve navigator efficiency and maintain image quality and strains in children.

## Methods

### Feedback videogame

**Fig. 2:**
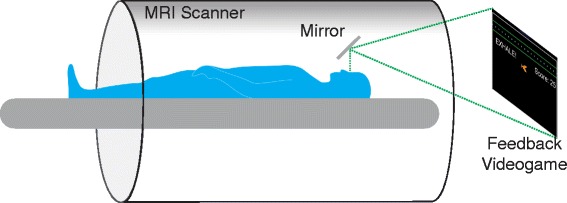
Feedback videogame was shown to children during CMR with an angled mirror and MR-compatible projector

A navigator feedback videogame (FG), called “Bubble Gulp”, was developed using MATLAB (The Mathworks Inc, Natick, MA). Each frame of the navigator image provided within the Siemens Syngo user-interface (Siemens Healthcare, Erlangen, Germany) (Fig. 1a) was captured using an Epiphan DVI2USB 3.0 (Epiphan Systems Inc., Palo Alto, California) frame grabber and processed in real-time during CMR to yield a kid-friendly representation of the diaphragm position (Fig. 1b). Navigator image processing was performed using an externally connected laptop running Windows 7 with an Intel Core i7 processor and 16 GB of RAM. The FG interface was then projected to the participant in the scanner using an angled mirror and a magnetic resonance compatible projector (Fig. 2).

The diaphragm position relative to the acceptance window (Fig. 1a) was represented by the vertical position of a fish character relative to parallel green lines containing scrolling dots, representing bubbles (Fig. 1b). The objective of the game was to control the fish’s vertical position, which was updated with each navigator pulse, so it would “gulp” bubbles and acquire points. To incentivize slow, stable breathing, point values increased as the fish spent more time within the green lines, instead of frequent short-duration breath-holds. However, prior to any use of the FG, children were instructed to not hold their breath for an uncomfortable amount of time and to breathe when needed. Finally, the FG interface displayed text to instruct children how to adjust their breathing in order to place the fish in between the green lines (Fig. 1b).

### Participants

Fifty children with no significant past medical history were recruited to participate in the study. Participants were recruited from the broader clinical community based out of our university medical center using a wide range of participant recruitment services provided by the University of Kentucky Center for Clinical and Translational Science. All participants were screened with a 12-lead ECG prior to imaging to rule out arrhythmias. The local Institutional Review Board at the University of Kentucky approved the study protocol and all participants and legal guardians provided written informed consent or assent.

### Imaging

All imaging was performed using a 3T Siemens Tim Trio (Siemens Healthcare, Erlangen, Germany) with a 6-element chest coil and a 24-element spine coil. For each participant, navigator-gated 2D spiral cine DENSE CMR [1, 11] images from mid-ventricular, 4-chamber, basal, and apical image orientations were separately acquired with no feedback (NF) and then while using the FG. No instructions regarding breathing were given for the NF acquisitions, thus participants were allowed to breathe naturally. Between acquisitions with NF and those with the FG, each participant underwent two 30-heartbeat practice scans to familiarize himself or herself with the FG.

DENSE imaging parameters included: number of spiral interleaves = 12, interleaves per beat = 2, FOV = 360 × 360 mm^2^, pixel spacing = 2.8 × 2.8 mm^2^, slice thickness = 8 mm, TE = 1.4 ms, TR = 17 ms, variable flip angle = 20°, displacement encoding = 0.06 cyc/mm [12], through-plane dephasing = 0.08 cyc/mm [13], CSPAMM echo suppression [14], view sharing and a dual-navigator strategy [15] with an acceptance window size of ± 3 mm. For each cardiac cycle, the navigator echo occurred immediately after data acquisition. The dual-navigator strategy required the diaphragm position to be within the acceptance window for both the preceding *and* current cardiac cycles in order for data to be accepted. Prospective ECG gating was performed and 11–25 cardiac phases were acquired depending on participant heart rate. As a result of the imaging parameters, each complete image acquisition required 38 heartbeats that satisfied the navigator gating criteria.

Due to erratic respiratory patterns or participant movement, image acquisition can be difficult to complete in children in a reasonable amount of time with NF. As scan session duration increases, the likelihood of patient movement also increases, so we defined criteria for maintaining a target scan protocol duration of 30 min. We defined image acquisition as incomplete (data not acquired) following 192 heartbeats without a completed image acquisition. Progressing past 192 heartbeats for a 38-heartbeat scan is equivalent to achieving less than 20 % navigator efficiency, which is worse than previously reported NF values [4–7]. Once any NF image acquisition was marked as incomplete, we proceeded to the FG acquisitions. If a participant moved, the number of acquired image orientations was reduced from four (mid, 4ch, base, apex) to two (mid, 4ch) to ensure at least two images were acquired with NF and FG.

### Calculation of cardiac strains from DENSE

DENSE images were analyzed using *DENSEanalysis* (denseanalysis.com), a custom, open-source MATLAB (the Mathworks Inc, Natick, MA) software. To delineate the myocardium, endocardial and epicardial boundaries were manually drawn on the DENSE magnitude image using an end-systolic and end-diastolic frame. The motion field was reconstructed using a simplified analysis technique [16]. Using manual selection of seed points, which indicated unwrapped phase data, a path following algorithm was used to unwrap the displacement-encoded phase data. Temporal fitting and spatial smoothing was applied to the resulting Lagrangian displacements as previously described [17].

Two-dimensional segmental Lagrangian strains were quantified from the smoothed trajectories over the entire cardiac cycle. Radial and circumferential strain was computed for 6 myocardial segments of the short-axis images and longitudinal strain was computed from the long-axis images. The strain curves of all the cardiac segments were averaged into a single average curve. Global peak strain was quantified by averaging the strain curves from each slice and finding the resulting peak strain of this curve. When computing peak longitudinal strain, pixels within 10 % of left ventricular longitudinal length of the most basal and apical regions were excluded due to increased noise typically observed in the strain curves in those regions. Peak strain was defined as a positive for thickening (radial strain) and negative for shortening (circumferential and longitudinal strain).

### Analysis

This study measured navigator efficiency and heart rate during image acquisition and used image signal-to-noise ratio (SNR) of the end-systolic DENSE magnitude image as a measure of image quality. Navigator efficiency was defined as the ratio of the number of heartbeats for which image data were accepted to the total number of heart beats required to complete the image acquisition. To compare image quality, signal to noise ratio (SNR) was calculated for each cardiac phase of each DENSE magnitude image. SNR was computed from the average myocardium signal and the standard deviation of the signal (noise) within an area with no signal (free from tissue and imaging artifacts). Due to the Rician distribution of the MR signal, corrections were applied to the measured standard deviation (*σ*_*M*_ in Eq. 1) and measured myocardial signal (*M* in Eq. 2) to compute the true SNR [11, 12, 18]. The SNR was defined as the ratio of the true myocardial signal to the true standard deviation.

1 $$ \sigma = \sqrt{\frac{2}{4-\pi }}*{\sigma}_M \approx 1.526*{\sigma}_M $$

2 $$ S = \sqrt{M^2-{\sigma}^2} $$

For incomplete NF image acquisitions (satisfied stoppage criterion), navigator efficiency and heart rate measurements were computed based on the partial data that were acquired.

### Training

Off-scanner training has been used by other investigators to ensure participants are comfortable and understand a navigator feedback interface before entering the magnet [5]. We wanted to determine the efficacy of off-scanner training with the FG on navigator efficiency, image quality, and heart rate. Thus, 30 of the 50 enrolled participants were randomized into equal groups to either receive extensive off-scanner training or no off-scanner training prior to scanning; thus, the groups were referred to as ‘trained’ and ‘untrained.’ As mentioned above, all subjects (including trained and untrained participants) underwent minimal training *in the scanner*, which was defined as two 30-heartbeat practice scans prior to FG acquisitions. The remaining 20 participants also received off-scanner training, but they were not included within the trained subgroup for analysis because they were not randomized to this treatment.

Each trained participant was introduced to the FG using an MRI simulator prior to the formal study. The MRI simulator utilized a PrimeSense Carmine 1.09 (PrimeSense, Tel Aviv, Israel) 3D camera to precisely measure the chest wall and abdomen excursion as a proxy for diaphragm translation [19, 20]. Each participant had to complete goal-based training before advancing to CMR scanning. Training time was recorded for all trained participants. The training protocol is described in detail in Additional file 1.

### Statistics

Statistical analyses were completed using R version 3.2.2 (R Foundation for Statistical Computing, Vienna, Austria). All continuous measurements were reported as mean ± standard deviation. Navigator efficiency, SNR, heart rate, and global left ventricular strains were tested for normality using a Shapiro-Wilk test. Average navigator efficiency, SNR, heart rate, and strain were compared between NF and FG acquisitions using a paired student’s *t*-test or Wilcoxon Signed-Rank test when appropriate, and compared between untrained and trained groups using a student’s *t*-test or Mann-Whitney *U* test when appropriate. To determine whether age had an effect on navigator efficiency, age was correlated with navigator efficiency for both NF and FG acquisitions.

## Results

Fifty-six children were prospectively enrolled. Six children were excluded from the study due to either being uncomfortable in an MRI scanner, having premature ventricular contractions, having ECG-monitoring equipment fail, or consistently moving during scanning. Thus, this study reported data on 50 children (Age: 14 ± 3 years, 48 % female) with no significant past medical history, which included a subset of 30 children randomized to either the off-scanner trained (*n* = 15; Age: 15 ± 3 years, 47 % female) or untrained (*n* = 15; Age: 13 ± 3, 66 % female) groups. All trained participants successfully completed off-scanner training and the mean training duration was 11 ± 2 min. The prescribed stoppage criterion for the NF scans was met in 11 cases, resulting in fewer completed NF images for those participants. Additionally, four participants moved during scanning, which included two during NF scans and two during FG scans, resulting in the completion of the abridged imaging protocol, as described in the methods.

### Navigator efficiency

**Fig. 3:**
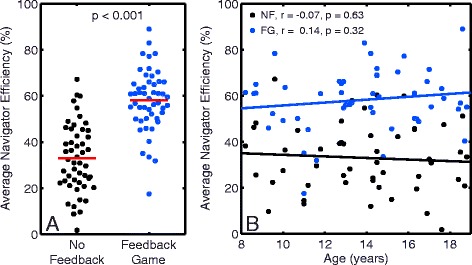
**a** Average navigator efficiency for No Feedback and Feedback Game image acquisitions. Use of the feedback game significantly increased navigator efficiency compared to no feedback. The solid red line indicates the mean of each group. **b** Average navigator efficiency vs age for No Feedback (NF) and Feedback Game (FG) image acquisitions. There was no correlation between navigator efficiency and age for either no feedback (*r* = -0.07, *p* = 0.63) or feedback game (*r* = 0.14, *p* = 0.32) acquisitions. The solid lines indicate the line of best fit for each group

**Fig. 4:**
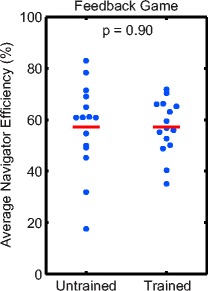
Average navigator efficiency for Off-scanner Trained and Untrained groups. There was no significant difference in navigator efficiency between untrained and off-scanner trained groups for feedback game acquisitions. The solid red line indicates the mean of each group

Using the FG significantly improved average navigator efficiency compared to NF (58 ± 13 % vs 33 ± 15 %, *p* < 0.001, Fig. 3a). Average navigator efficiency was not correlated with age for either NF or FG image acquisitions (*r* = −0.07, *p* = 0.63; *r* = 0.14, *p* = 0.32, Fig. 3b). There was no significant difference in average navigator efficiency between untrained and off-scanner trained groups for FG image acquisitions (57 ± 17 % vs 57 ± 11 %, *p* = 0.90, Fig. 4).

### SNR

**Fig. 5:**
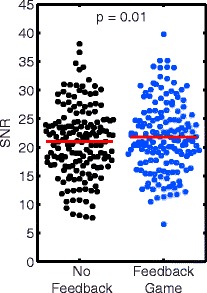
SNR for all No Feedback and Feedback Game images. Use of the feedback game resulted in significantly increased SNR compared to no feedback

Use of the FG significantly improved SNR compared to NF (22 ± 6 vs 21 ± 6, *p* = 0.01, Fig. 5). There was no significant difference in SNR between untrained and off-scanner trained groups for FG images (22 ± 6 vs 21 ± 6, *p* = 0.77).

**Table 1:** Average Heart Rate for Off-scanner Trained and Untrained groups

Heart Rate (bpm)	Trained		*p*-value	Untrained		*p*-value
	No Feedback	Feedback Game		No Feedback	Feedback Game	
Mean	72 ± 13	76 ± 16	0.03	72 ± 9	78 ± 9	<0.001
Standard Deviation	6.9 ± 5.0	5.7 ± 2.4	0.80	5.3 ± 2.4	6.0 ± 2.0	0.17

### Heart rate

**Fig. 6:**
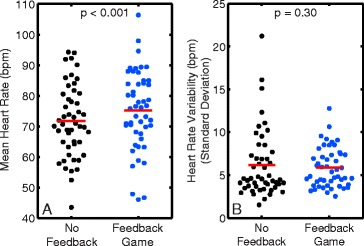
Mean and standard deviation of heart rate for No Feedback and Feedback Game acquisitions. **a.** Use of the feedback game resulted in significantly higher heart rate compared to no feedback. **b.** There was no significant difference in standard deviation of heart rate between no feedback and feedback game acquisitions. The solid red line indicates the mean of each group

On average, heart rate during FG scans was slightly higher than NF acquisitions (75 ± 13 vs 72 ± 12 bpm, *p* < 0.001, Fig. 6a), but there were no differences in the standard deviation of heart rate (5.9 ± 2.2 vs 6.1 ± 3.9 bpm, *p* = 0.30, Fig. 6b). Heart rate was similarly elevated during FG acquisitions in both the untrained and off-scanner trained groups compared to NF acquisitions (*p* < 0.001 and *p* = 0.03, respectively, Table 1).

### Strain

Global circumferential and radial strains derived from FG acquisitions were slightly lower in magnitude compared to NF acquisitions (−16 ± 2 % vs −17 ± 2 %, *p* < 0.001; 40 ± 10 % vs 44 ± 11 %, *p* = 0.005, respectively, Table 2). There were no differences in longitudinal strain between NF and FG acquisitions (−13 ± 2 % vs −13 ± 2 %, *p* = 0.38).

**Table 2:** Global peak strain results for NF and FG scans

	No Feedback	Feedback Game	*p*-value
Circumferential Strain (%)	−17 ± 2	−16 ± 2	<0.001
Radial Strain (%)	44 ± 11	40 ± 10	0.005
Longitudinal Strain (%)	−13 ± 2	−13 ± 2	0.38

## Discussion

Feedback of the diaphragm position during CMR has been shown to improve navigator efficiency in adults [5, 6]. This study explored how the use of a feedback game (FG) affects navigator efficiency compared to traditional no-feedback (NF) acquisitions *in children*. The results of the study showed that, compared to NF, using the FG resulted in 1) substantially improved navigator efficiency (from 33 to 58 %); 2) slightly improved SNR; 3) slightly higher mean heart rate; and 4) slightly lower global strain magnitudes. Importantly, these results were not affected by the use of an off-scanner training protocol, which suggests that lengthy, robust training (11 min in our protocol) does not need to be a part of the clinical/imaging workflow for this interface.

### Navigator efficiency

Navigator efficiency was improved from 33 to 58 % by using a FG in children (Fig. 3a). This increase in navigator efficiency led to a 43 % reduction in the number of heartbeats required to complete a scan. Studies have shown that feedback of the diaphragm position during CMR results in a more reproducible breath-hold position [5, 8, 21], which can lead to improved navigator efficiency. Previous CMR studies have reported that NF navigator efficiencies can vary from 20 to 45 % in adults [4–7], and we found a comparable NF navigator efficiency of 33 % in children using a conservative dual-navigator acceptance strategy. Visual feedback of the diaphragm position has been shown to improve end-expiratory navigator efficiency from 45 to 56 % [6] and from 42 to 71 % with the addition of supplemental oxygen [5] leading to a 20 % and 41 % reduction in the number of required heartbeats, respectively. With the use of the FG, we found a slightly better improvement of navigator efficiency from 33 to 58 % in children without the use of supplemental oxygen. Average navigator efficiency was not correlated with age (Fig. 3b). Therefore, children ages eight and older should be able to utilize the FG to effectively improve navigator efficiency compared to conventional NF acquisitions.

Extensive off-scanner training using an MRI-simulator was not necessary to achieve the observed improvement in navigator efficiency using the FG. Instead, the subjects with minimal training immediately prior to data acquisition had equivalent efficiency as their extensively-trained counterparts. While this finding might suggest that the chest wall excursion-based training method was ineffective, it is more likely that the intuitive interface design was easy to learn and therefore the children did not require much training. Importantly, the two 30-beat practice scans provided some degree of training in both cases, which is intuitively necessary. Future efforts can optimize that practice time to provide the needed minimal training in the most efficient manner.

### SNR

We found that using the FG slightly improved the SNR of the end-systolic magnitude images of our spiral DENSE sequence by 5 % compared to NF for all images combined (*p* = 0.01, Fig. 5). This finding contrasts with previous studies, which reported image quality score using 2 expert reviewers and found that the use of diaphragmatic feedback maintained image quality compared to NF acquisitions [5, 6]. The difference in image quality is likely sequence dependent. The previous studies were performed using steady-state free precession. Additionally, it is likely that quantitative measurement of SNR is more sensitive at detecting differences in image quality compared to subjective image scoring by expert reviewers.

### Heart rate

A potential negative finding of this study was the slight increase in heart rate observed with the use of the feedback game. To be clear, this difference did not represent an increase in heart rate variability—as evidenced by the comparable standard deviation values—but simply a higher baseline value. Such findings are not unprecedented, as a previous CMR study found a mean heart rate increase of 5 beats/min with use of navigator feedback in adults (compared to our 3 beats/min), and similarly no differences in heart rate variability between NF and navigator feedback [5]. A likely reason for this difference is the longer breath-holds performed during the FG, which could have increased the heart rate, compared to relaxed breathing during NF. Another mechanism could be related to stimulation and adrenaline associated with playing the game, compared to the relaxed, passive state associated with NF.

The importance and implications of this potential heart rate difference likely depends on the imaging application. While it may mean very little for purely anatomic evaluations, functional measures, such as strains, may be affected by changing loading conditions and contractility [22]. To counteract such effects, if undesirable, patients could be coached to relax when playing the game and to not be too competitive. The design of the game could be modified to enforce such behavior; for example, by programmatically requiring the participant to inhale/exhale after a fixed period of time, or instructing him/her to periodically take a series of relaxed breaths between cycles of breath-holding.

### Strains

We observed small, but statistically significant decreases in global circumferential and radial strains with use of the FG, compared with NF. There was, however, no difference in longitudinal strain. While these findings warrant further study and consideration, the clinical relevance of such small differences (1 % for circumferential strain, 4 % for radial strain) is likely minimal as they are smaller than previously observed inter-test (±2.0 % for circumferential, ±13 % for radial) and inter-observer (±1.4 % for circumferential, ±14 % for radial) 95 % limits of agreement for DENSE [11, 12].

### Clinical implications

Importantly, the equipment needed to utilize the FG is minimal and does not directly integrate into an imaging sequence; it connects externally to the scanner user interface. Due to the minimal equipment needed and non-invasive connection to the MRI scanner, we anticipate that the FG system can be easily adopted at research and clinical sites that perform CMR navigator gating, especially in children.

Since navigator efficiency can be increased from 33 to 58 %, leading to reduced acquisition times, use of the FG can help improve the clinical feasibility of advanced imaging techniques. While reducing the acquisition time would likely be the most common use of increased navigator efficiency from the FG, the saved time could be allocated to improve image spatial or temporal resolution [5]. Importantly, pre-scan training was not necessary for navigator efficiency improvement with our system, so clinical and research sites would not have to invest in an MRI simulator environment or spend significant time training children. Navigator feedback has been shown to reduce acquisition time in adults [5], thus, the use of the FG will likely work well in adults also.

Since we only acquired DENSE images for this study, the specific findings are only definitively relevant for DENSE. However, it is reasonable to expect that these findings are generalizable to many other CMR acquisitions that utilize a respiratory navigator. Possible exceptions include higher resolution applications, such as coronary MR angiography, which may be more sensitive to registration issues. Further study is needed to test this technique for these applications.

### Comparison with previous work

A previous study presented a respiratory biofeedback game and continuously adaptive windowing strategy (CLAWS) to increase navigator efficiency of imaging the thoracic aorta. The authors reported an increase in efficiency in that study from 45 to 56 % in adults [6], which represents a smaller magnitude of improvement (25 % vs. 11 %) but a similar end result (58 % vs. 56 %) compared to our study. Although the two studies are similar, there are distinct differences in design. Most notably, the previous study was in adults; whereas we exclusively focused on children, based on their limited ability to breath-hold and thus potentially greater need for respiratory navigated sequences. Additionally, the previous study modified their pulse sequence to allow acquisition of multiple navigator echoes, likely providing a smoother game experience. We did not modify our cine pulse sequence in our evaluation—we had a single navigator echo per cardiac cycle—in order to ensure more general clinical applicability. Collectively, these studies demonstrate the potential utility of user-friendly interfaces for improving efficiency and image quality of cardiovascular imaging sequences using a respiratory navigator in a broad array of patients.

### Limitations

This study used a dual-navigator strategy when performing image acquisition. Dual-navigator strategies have stricter data acceptance criteria compared to previously used single-navigator strategies [1], and, given the same imaging parameters, will likely result in lower navigator efficiencies. However, a previous study using a single-navigator strategy with navigator feedback reported similar navigator efficiency results compared to our study. Therefore, the use of the FG with a single-navigator strategy will likely have similar results to this study except that both NF and FG acquisitions may have improved navigator efficiency compared to a dual-navigator strategy.

The respiratory navigator gating sequence used in this study only measured the diaphragm position once per cardiac cycle. This low refresh rate can make fine control of the diaphragm position more challenging, especially for participants who may have lower heart rates. Increasing the number of navigator echoes per cardiac cycle could therefore improve performance, but such modifications may not be possible for all sequences, as is the case for DENSE. Furthermore, even with this limitation, we still found substantial improvement in navigator efficiency when using the FG compared to NF acquisitions.

Due to the randomization of the participants into the trained and untrained groups, there was no attempt to balance age between groups. Therefore, the average trained participant was about 2 years older than the average untrained participant. We found that there was no difference in FG navigator efficiency between trained and untrained participants. Even though there was an age difference between trained and untrained groups, there was no correlation between age and navigator efficiency with the FG (Fig. 3b); thus, the results of the study apply to all children aged eight to eighteen.

In order to accurately assess the NF navigator efficiency as it would be in the clinical setting, we did not want to influence the children’s natural breathing pattern. In particular, we did not want the breathing pattern performed during the FG acquisitions to influence the NF breathing pattern. Therefore, NF acquisitions were always performed before FG acquisitions. Since the order of NF and FG acquisitions was not randomized, this may have affected the results as participants may have become more comfortable as they spent more time in the MRI scanner. However, performing this randomization likely would have resulted in similar conclusions and we feel that it was important to accurately measure the navigator efficiency of the NF acquisitions.

Due to the potential for patient movement or erratic breathing patterns, we utilized a stoppage criterion to attempt to maintain a 30 min protocol length. We observed eleven cases which satisfied stoppage criterion and four cases of patient movement (one which also satisfied stoppage criterion). In these participants, we estimated navigator efficiency, SNR, and heart rate from fewer acquisitions than the remaining participants. However, since we used all of the data that we did acquire for each participant, the computed values are appropriate.

The two 30-heartbeat practice scans were not included in the computation and analysis of navigator efficiency for the FG technique. Their inclusion would only slightly decrease the reported gains in efficiency (for example, if we used the FG to acquire 300 heart beats of actual data, the reduction in scan time would change minimally from 43 to 37 % after accounting for the two practice scans); however, it must be noted that the selection of those practice parameters was arbitrary and not optimized. In reality, less training is likely required to familiarize the subject with the interface, so factoring this specific training design into the analysis is not critical.

We performed this study in children with no significant past medical history. While we did attempt to recruit from a broad clinical population using recruitment services at our Center for Clinical and Translational Science, the population we ultimately studied may not be entirely representative of a standard pediatric clinical population that would routinely undergo cardiac MRI. For example, approximately 25 % of patients with tetralogy of Fallot may have learning and behavioral difficulties [23], which may impair their ability to benefit from the feedback game. It is therefore reasonable to expect that the true benefit of the feedback game in a standard clinical population will be smaller than what was measured in the current study, but still better than what can be expected without the use of feedback. Even if only half of the patients benefit to the extent shown in the current study, the overall navigator efficiency for the clinical population as a whole would still increase from 33 % efficiency to 46 % efficiency (a 38 % relative benefit). Future research will seek to evaluate this in further detail as we implement the feedback game during routine clinical workflows.

## Conclusion

Use of a respiratory navigator feedback game designed to engage children during navigator-gated CMR improved navigator efficiency in children from 33 to 58 %. This improved efficiency reduces the number of heartbeats and corresponding scan durations by 43 %, and is also associated with a 5 % increase in SNR for spiral cine DENSE. Pre-scan training on how to use the feedback game is not necessary to achieve the improvement in navigator efficiency. These findings should generalize to all CMR acquisition sequences that utilize a respiratory navigator.

## Additional file

Additional file 1: Training Protocol and Survey Responses. (PDF 51 kb)

## Abbreviations

CMR: Cardiovascular magnetic resonance
DENSE: Displacement encoding with stimulated echoes
ECG: Electrocardiogram
FG: Feedback game
MRI: Magnetic resonance imaging
NF: No feedback
SNR: Signal-to-noise ratio
