# Supplementary material for: An interactive videogame designed to improve respiratory navigator efficiency in children undergoing cardiovascular magnetic resonance
Source: J Cardiovasc Magn Reson. 2016 Sep 6;18(1):54. doi: 10.1186/s12968-016-0272-z (PMC5012042; doi:10.1186/s12968-016-0272-z)

Supplemental Material

Training Protocol

The goal-based training protocol was as follows: First, the children were instructed to perform 3 sequential end-expiratory breath-holds to determine the optimal location for the acceptance window. Then the children were instructed to complete 9 levels of the FG, which progressed in difficulty. Difficulty was increased by either 1) decreasing the acceptance window size or 2) increasing the time delay between chest excursion recording and fish location update. Because the navigator gating sequence only measures the diaphragm position during each heartbeat, children with slower heartbeats may experience “delays” between diaphragm movement and fish location update. In order to complete each level, the children had to acquire 100 points. If all bubbles were acquired in a row with no breaks, each level could be completed in ~33 seconds.

Survey Responses

In order to formally measure the enjoyment and response of the children playing the Feedback Game, we asked the children to fill out a post-scan survey that consisted of 7 questions. Those questions and responses are listed below. In general, most participants 1) found Bubble Gulp to be easy to play; 2) enjoyed playing Bubble Gulp; 3) thought they were generally getting better as they played; 4) thought training was/would have been somewhat helpful; 5) had no comments on how to improve ‘Bubble Gulp’; 6) enjoy playing videogames; and 7) play videogames daily.

1. How easy was playing Bubble Gulp?

1	2	3	4	5
Really easy	Easy	Neither easy nor difficult	Difficult	Really difficult

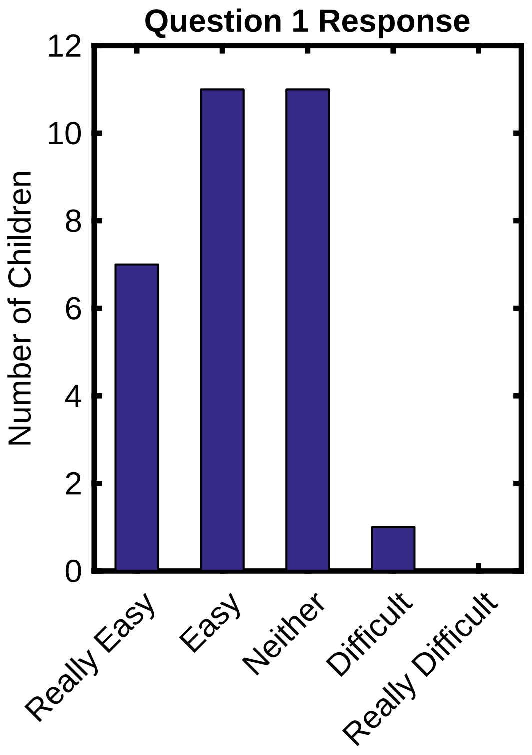

2. How much did you enjoy playing Bubble Gulp?

1	2	3	4	5
Really enjoyed it	Enjoyed it	Neither	Did not enjoy it	Really didn't enjoy it

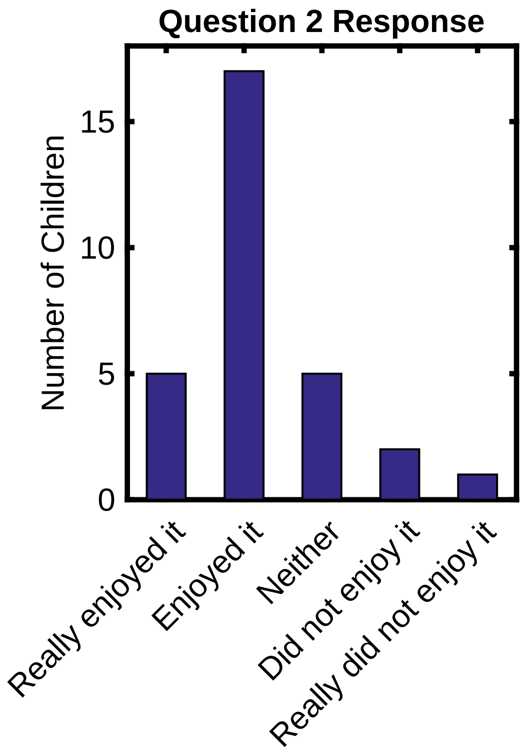

3. Did you think you were getting better, stayed the same, or were getting worse as you were playing Bubble Gulp at the end of the study compared to when you first tried it?

1	2	3
Better	Stayed the Same	Worse

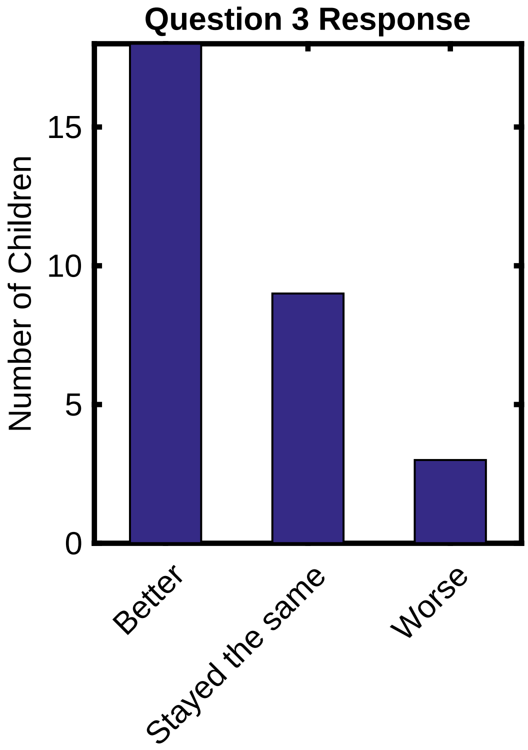

4. We have a pretend MRI scanner where you can learn to play Bubble Gulp before getting into the actual MRI scanner. Do you think using this pretend MRI scanner first would have been/was:

1	2	3	4
Very Helpful	Somewhat Helpful	Not helpful	A total waste of time

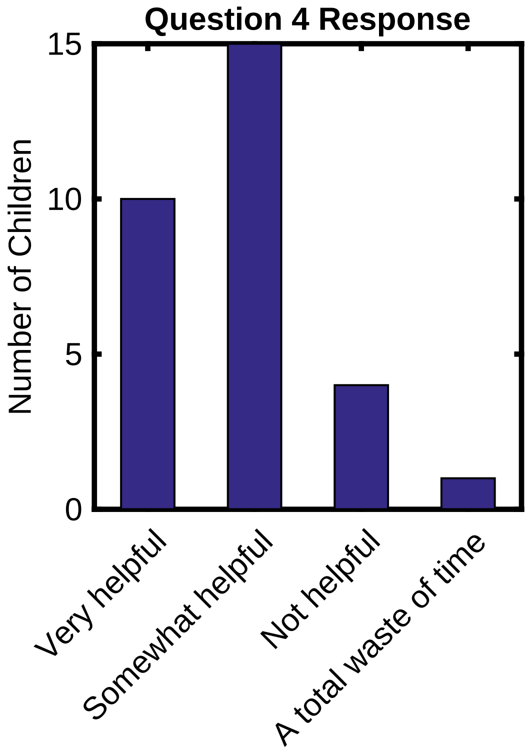

5. Do you have any comments on how to improve Bubble Gulp?
   - Mostly “None”
   - “Make the fish pink”
   - “Make the lines further a part on the screen”
   - “Liked the simple concept and how could control with breathing”
   - “Reverse direction of fish movement with breathing”
   - “Make not as glitchy, (make smoother)”
   - “Make lines move to more comfortable spot to breathe in”
     - This subject moved before their last scan

6. How much do you enjoy playing videogames?

1	2	3	4	5
Really enjoy	Enjoy	Neither	Do not enjoy	Really don't enjoy

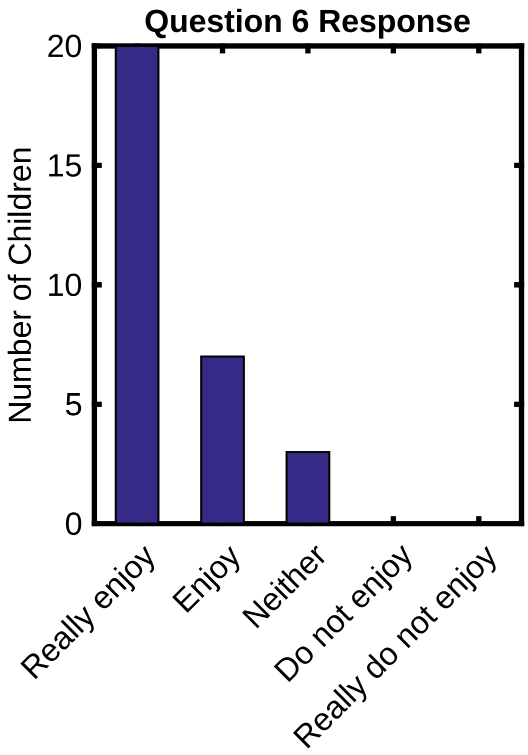

7. How often do you play videogames?

1	2	3	4	5
Daily	2-3 times per week	Weekly	1-2 times per month	Seldom to never

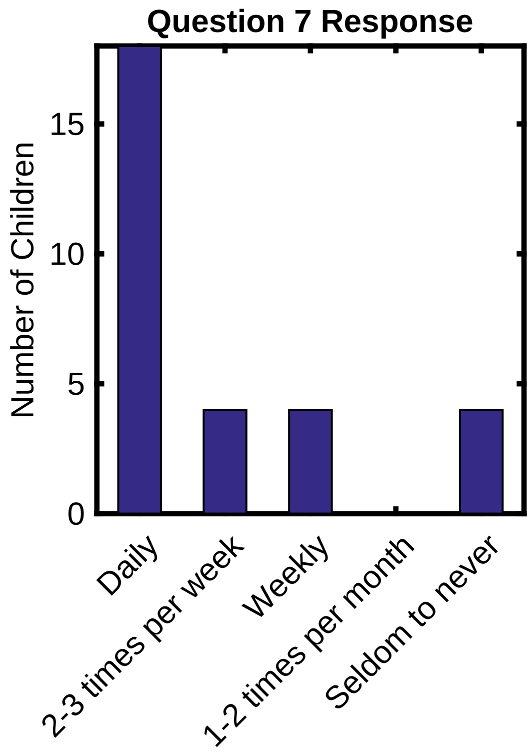

Supplement: Additional file 1: — Training Protocol and Survey Responses. (PDF 51 kb) [file 12968_2016_272_MOESM1_ESM.pdf]
